# Researchers Face Challenges Devising a PFAS-Free Future

**DOI:** 10.1021/acscentsci.5c00389

**Published:** 2025-03-11

**Authors:** XiaoZhi Lim

From electronic door handles to massaging seats, there’s no shortage of flashy upgrades in new cars. But Volkswagen’s electric ID.3 series ushered in a change that most drivers may have missed. Since 2020, automakers Volkswagen, Audi, and Škoda Auto have manufactured over 700,000 vehicles that use carbon dioxide as a refrigerant to keep both engines and cabins at just the right temperatures.

**Figure:**
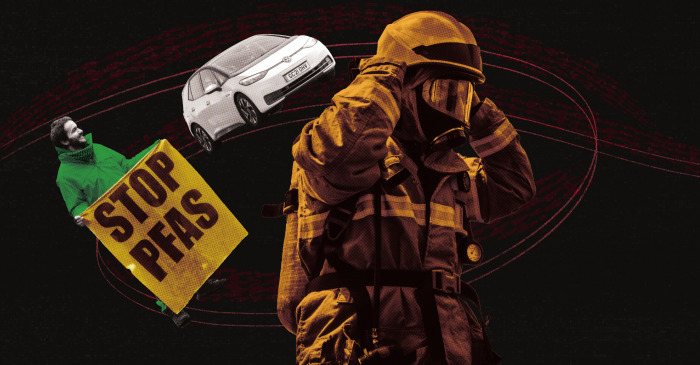
Madeline Monroe/C&EN/Shutterstock/Alamy

The CO_2_ circulating in these cars, also known as R744 among refrigerant makers, bucks a century-long trend. Since the 1920s, most refrigerants have depended on molecules that contain fluorine. This small and electron-loving element makes refrigerant molecules chemically stable and nonflammable; it also keeps them light enough to be free-flowing gases and liquids at the pressures used in refrigeration equipment. Indeed, a vast range of modern technologies beyond refrigeration depend on fluorine-bearing substances that can deliver the right combination of properties.

But since the 1990s, the public has learned firsthand that some fluorine-bearing molecules called per- and polyfluoroalkyl substances (PFAS) come with a slew of problems. Environment-wise, some fluorinated refrigerants are long-lasting greenhouse gases. And health-wise, communities living near fluorochemical processing plants experience higher rates of disease. In one of the most high-profile cases, DuPont and Chemours paid residents of Parkersburg, West Virginia, $670 million in 2017 to settle thousands of lawsuits related to PFAS in drinking water. Residents reported higher rates of two types of cancer, among other ailments.

As public and regulatory pressure mounts, users are increasingly looking for alternatives to help them go fluorine-free. Already, companies, states, and whole countries—for example, France in February—have banned PFAS in household products, including cosmetics and carpets, and the broader European Union is currently evaluating a sweeping proposal to ban PFAS. Seeing the writing on the wall, some recreational users have proactively dropped fluorochemical-based products. Since 2020, for example, US Ski and Snowboard has banned fluorocarbon ski waxes in domestic races.

But adding a few seconds to your ski run is less of a concern than driving without heat in below-freezing temperatures. If regulators around the world start banning PFAS broadly, many users could be left stranded, says materials scientist Kevin Golovin from the University of Toronto. “They may be forced to use nonfluorinated [chemistries], and that’s where new innovation in this space is required.”

While innovation would go a long way, whether these users can successfully find suitable alternatives to fluorine-based materials might depend also on designing devices or usage practices to accommodate material changes. “We have to come to terms with the fact that if we want to replace a ‘forever polymer,’ it’s going to have less ‘forever’ character, or in other words, reduced stability in certain environments,” says Justin Kennemur, a polymer chemist at Florida State University.

## Under pressure

Among fluorochemistry applications, refrigeration units take the top spot when it comes to releasing pollution, according to an analysis by European regulatory agencies. As part of millions of home, commercial, and mobile air-conditioning units, refrigerants have long caused pollution problems when they leak from the products they’re in. Refrigerants of the early 20th century, such as the chlorofluorocarbons in many Freon products, were discovered to deplete Earth’s ozone layer, and the hydrofluorocarbons that replaced them were found to be potent greenhouse gases.

And newer refrigerants have come with new problems. The now-common refrigerant 2,3,3,3-tetrafluoropropene, or R1234yf, breaks down into a smaller PFAS, trifluoroacetic acid (TFA), which researchers are documenting at comparatively high concentrations in the environment. One study found that TFA comprised over 90% of the total concentration of 43 small PFAS molecules in German drinking-water sources.

Executives hope R744 will be a more sustainable substitution. “We chose R744 because of its environmental compatibility, so it’s a very good refrigerant in that regard,” Volkswagen engineer Felix Nowak-Walenta shared at a webinar organized by the Sweden-based nonprofit International Chemical Secretariat.

R744 really shines when it comes to electric cars, which gain some range when their batteries stay warm, says Nowak-Walenta. When used in cars’ heat pumps, the CO_2_ refrigerant works particularly well in cold climates, scavenging heat even at air temperatures below −15 or −20, when conventional refrigerants have trouble working at all.

But CO_2_ does come with challenges. Heat pumps using R744 operate at 130 bar (13 MPa), which is about four times the pressure required for R1234yf. That difference required stronger refrigerant lines and redesigning the heat pump.

Nowak-Walenta says it is not as radical as it may seem, though. “People think, ‘Oh, you need massive, massive refrigerant lines,’” he says, but engineers have already figured this problem out: lines for the two refrigerants can have the same wall thickness because R744 has a higher density than R1234yf. That property allows for walls of the refrigerant lines to be relatively thin despite the higher pressure demands. “It’s the same alloy; it’s the same thickness,” he says. “That’s really a misconception.”

Going fluorine-free in other refrigeration applications is a tougher sell. Internal combustion engines, which stay warm under the hood without a heater, do not get the same cost benefits as electric engines do from a CO_2_-based heat pump. The higher-pressure CO_2_ system is also more difficult to reproduce in larger-scale refrigeration applications. In fact, the EU’s draft proposal to ban PFAS anticipates that, barring a change in building safety codes, it could be nearly impossible to eliminate fluorinated refrigerants in home and commercial heating, ventilating, and air-conditioning (HVAC) systems. The proposal exempts those uses permanently.

## Repelling oils

After refrigerants, textiles represent the second-largest source of PFAS pollution. Fluorinated finishes are prized in the textile industry for their ability to repel both water—which keeps rain jackets, hiking boots, and car seats dry—and oils, coating scientist Golovin says.

Golovin’s team at the University of Toronto has been developing oleophobic coatings for glass or paper using polydimethylsiloxane (PDMS). Working at the nanoscale, Golovin and his colleagues developed a “hairbrush” design for their material, with PDMS polymer chains sticking out from a main polymer backbone like bristles on a brush.

**Figure:**
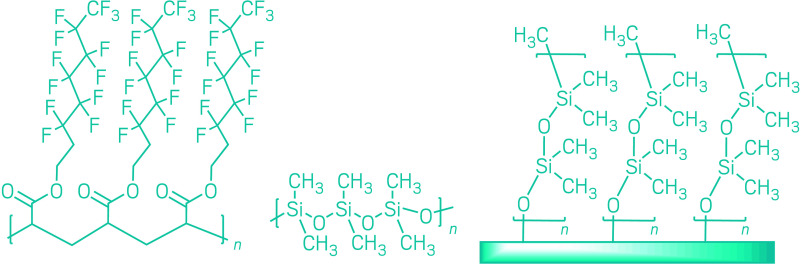
A typical fluorinated finish (left) repels oil and water from a surface thanks to fluorinated alkyl groups sticking out from a polymer backbone. Existing nonfluorinated finishes that repel water but not oil can be created with silicone-based coatings such as polydimethylsiloxane (center), but Kevin Golovin’s group at the University of Toronto is attempting to create materials with silicone chains that stick out from their substrate (right), with the hope that this will give them oil-repelling abilities too.

This coating’s oil-repelling abilities vary depending on the substrate. “If we’re just talking about glass, it can basically repel every liquid that all the fluorinated coatings can repel, and in some cases much better,” Golovin says. The coating works so well on glass that the researchers have begun looking into commercializing it to make fluorine-free smartphone screen treatments that can resist fingerprints.

But when the researchers tested the PDMS-based treatment on textiles, or on rough surfaces in general, it repelled fewer oils than fluorinated finishes and fared even worse after just one wash. This durability is not enough to be commercially useful, he says.

Just one chemical bond connects each PDMS polymer chain, or bristle, to the hairbrush backbone, so “if you break that one bond, that whole chain is removed,” Golovin says. His team is currently trying out chemical tweaks that might better attach each PDMS chain to the backbone and boost the coating’s staying power.

The current lack of a suitable fluorine-free yet oil-repellent textile finish may have already proved problematic for one of the most crucial applications of PFAS: fire-safety gear.

For decades, the outer shell of firefighters’ protective clothing, known as turnout gear, has been treated with fluorinated finish that can block many types of liquids, including oils, from soaking in, explains Bryan Ormond, a polymer and textile scientist at North Carolina State University. But with firefighters facing unusually high rates of cancer, unions and lawmakers have proactively worked to eliminate PFAS in firefighting gear, which included ditching the fluorinated finish.

“The manufacturers didn’t come up with a new finish for this; they went to finishes that are just water repellent,” Ormond says. That is a step down in performance because finishes that are just water repellent cannot repel oil, while finishes that can repel oil can also repel water. Water repellency is certainly important for turnout gear: when suits get wet, they weigh more and conduct heat faster to the person wearing them, and in the cold they freeze, immobilizing firefighters, he explains.

But oil repellency is also critical because firefighters might be exposed to diesel fuel, hydraulic fluid, motor oil, or even cooking oil when they respond to fires at auto body shops, gas stations, or restaurants.

In 2021, firefighting gear manufacturers across the US started switching to fabric treated with waxes or silicones, both of which contain alkyl groups on the surface, and now those kinds of coatings are the norm. But “you can’t repel hydrocarbons with a hydrocarbon surface,” Ormond says. Based on his team’s testing of turnout gear, Ormond has found that without the PFAS-based treatment, turnout gear could not repel oil or hydraulic fluid.

**Figure:**
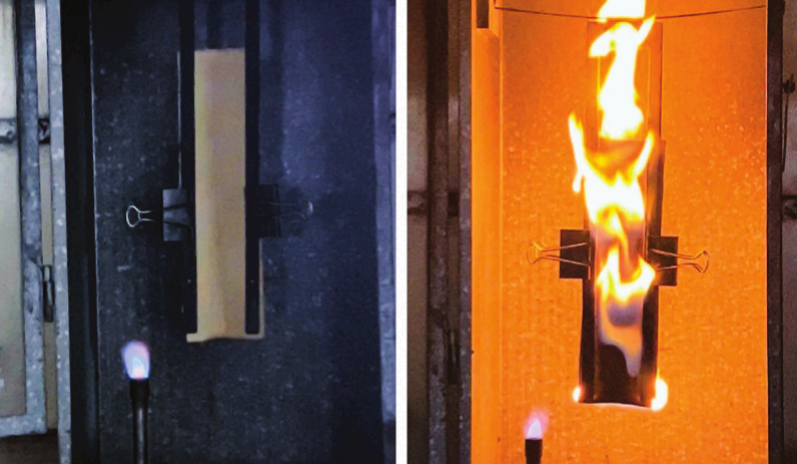
Researchers splashed diesel on fabrics with finishes used on the outer shell of firefighting gear. After exposing them to a flame, the fabric with a fluorinated finish (left) did not ignite, but the one with the nonfluorinated finish (right) lit and burned for 30–40 s after they removed the flame. Credit: Bryan Ormond/North Carolina State University.

To achieve a nonfluorinated oleophobic textile, Golovin expects that textile manufacturers will need to change not just the coating chemistry but also their choices of textile fiber and weave. “What we found is that you can’t really just hope for any textile to be oil repellent if you can’t use the fluorinated [coatings],” he says.

While waiting for materials scientists to catch up, Ormond has been giving presentations to fire chiefs to explain the differences between their old and new gear so that they can adjust how they approach and fight fires.

## Challenging an industry standard

Although PFAS are ubiquitous in consumer goods, most people have likely never encountered one of the most important products made with fluorine: the Nafion polymer. Manufactured by fluorochemical giant Chemours, the material has secured its place as an “industry standard” often used as a membrane to divide electrochemical systems such as fuel cells. Nafion membranes are also the go-to option for the chlor-alkali process, a workhorse industrial reaction largely responsible for the world’s sodium hydroxide and chlorine supplies.

As an ion-exchange membrane, Nafion allows cations but not anions to pass through. Besides having high ion conductivity, Nafion has mechanical strength and can withstand harsh chemical conditions. But as with other fluoropolymers, Nafion releases PFAS pollutants when it is manufactured and when it is used in applications.

For hydrogen fuel cell applications, Nafion conducts protons well because it has an extensive network of nanoscale water-filled channels lined with sulfonic acid groups, says Karen Winey from the University of Pennsylvania. For many years, chemists have believed that the electron-withdrawing fluorine atoms on Nafion’s polymer backbone turn the sulfonic acid groups into superacids, which was thought to be crucial for Nafion’s high proton conductivity. But now, “we can reproduce that without the fluorine,” she says.

Winey has been working on a fluorine-free ion-exchange membrane with Florida State’s Kennemur. Kennemur had spent years making a new analog of polystyrene: Instead of carrying a phenyl group on every other carbon of its backbone, the polymer that Kennemur’s team created bears a phenyl branch every five carbons. The researchers then created a sulfonated version of the polystyrene analog by adding sulfonic acid groups to the phenyl branches.

Intrigued, Winey asked for a sample, and Kennemur sent her a few grams. The ion-exchange membrane Winey’s team made using the polymer could conduct protons four times as well as Nafion does under the right conditions.

**Figure:**
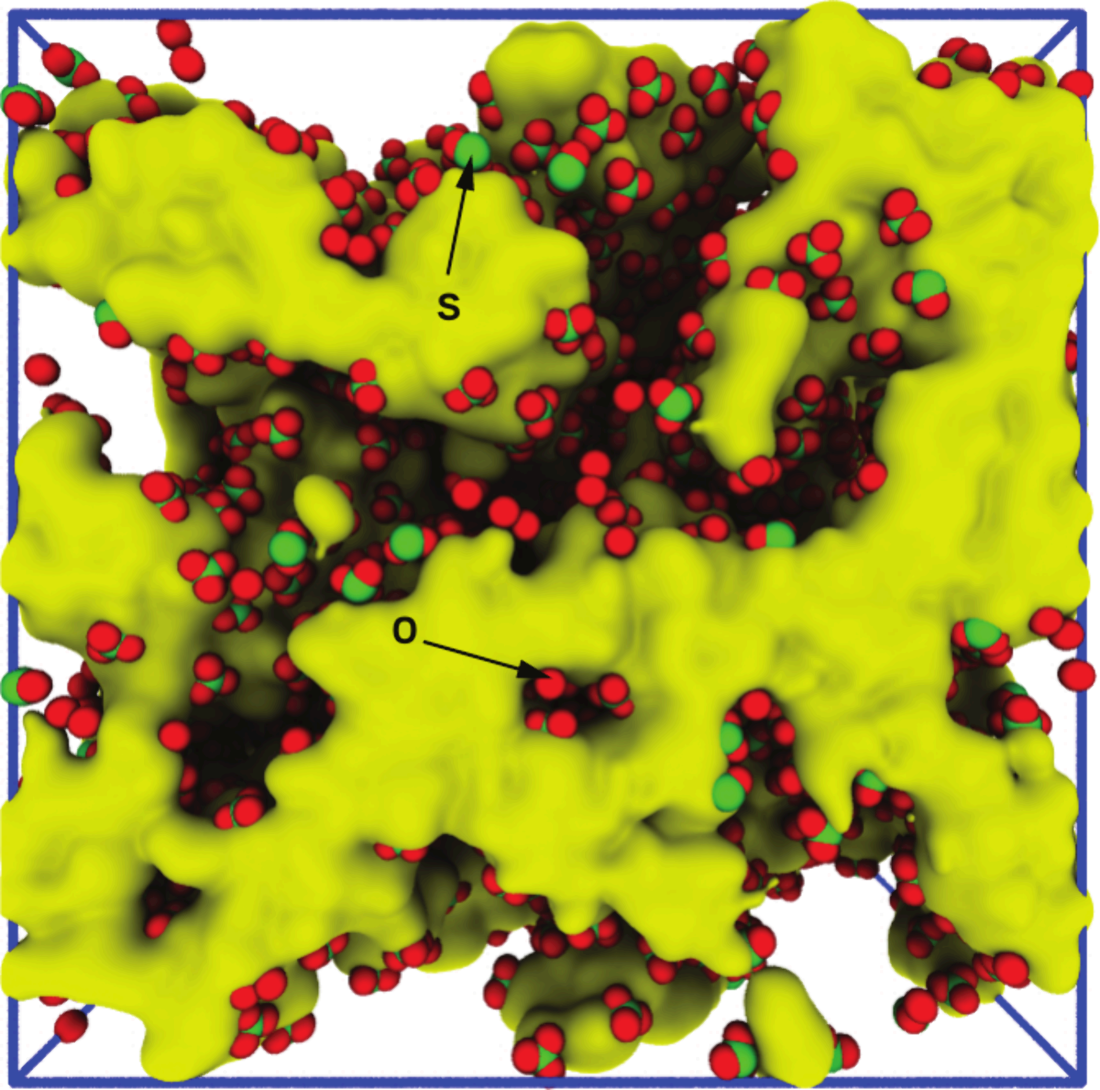
Sulfonate groups (red and green) arrange themselves densely along the interior of water channels in a fluorine-free ion-exchange membrane developed by Justin Kennemur, Karen Winey, and their collaborators at Sandia National Laboratories. These groups might be key to the material’s high ion conductivity. Credit: Amalie Frischknecht/Sandia National Laboratories/*Chemistry of Materials*.

Winey believes that, for high proton conductivity, flexibility in the polymer backbone might be more critical than the fluorine-derived superacid character because it allows the sulfonic acid groups to arrange themselves within the membrane’s water channels. “I think with a flexible backbone, there are a lot of materials that would do this,” she says.

The researchers also raised the membrane’s mechanical strength by reducing the number of sulfonic acid groups. But the team faces the biggest challenge yet as they embark on chemical stability tests. “I know that the material is not going to be as chemically stable as Nafion. I knew that when I made it,” Kennemur says.

While the material needs to be stable electrochemically and compatible with manufacturing processes used to build electrochemical devices, Winey says, new materials are rarely drop-in replacements; device optimization is also necessary. If engineers keep in mind that new fluorine-free membranes will likely break down faster, they could, for example, design devices with easily replaceable parts, Kennemur says.

Even if hydrocarbon polymers are not forever chemicals, they still last a long time. “If you’re not going to use fluoropolymers, hydrocarbon materials are the next best thing, and we’ve got a great one I think that’s worth pursuing,” Kennemur says.

## XiaoZhi Lim is a freelance contributor to

Chemical & Engineering News, *an independent news outlet of the American Chemical Society*.

